# Supplementary material for: The interactome of intact mitochondria by cross-linking mass spectrometry provides evidence for coexisting respiratory supercomplexes
Source: Mol Cell Proteomics. 2017 Dec 8;17(2):216–32. doi: 10.1074/mcp.RA117.000470 (PMC5795388; doi:10.1074/mcp.RA117.000470)
Supplement: Supplemental Data [file supp_RA117.000470_133922_0_supp_23978_fzffwf.zip › spectra_annotation/mito_spectra_annotation/98-1-6-1-8-1.pdf]

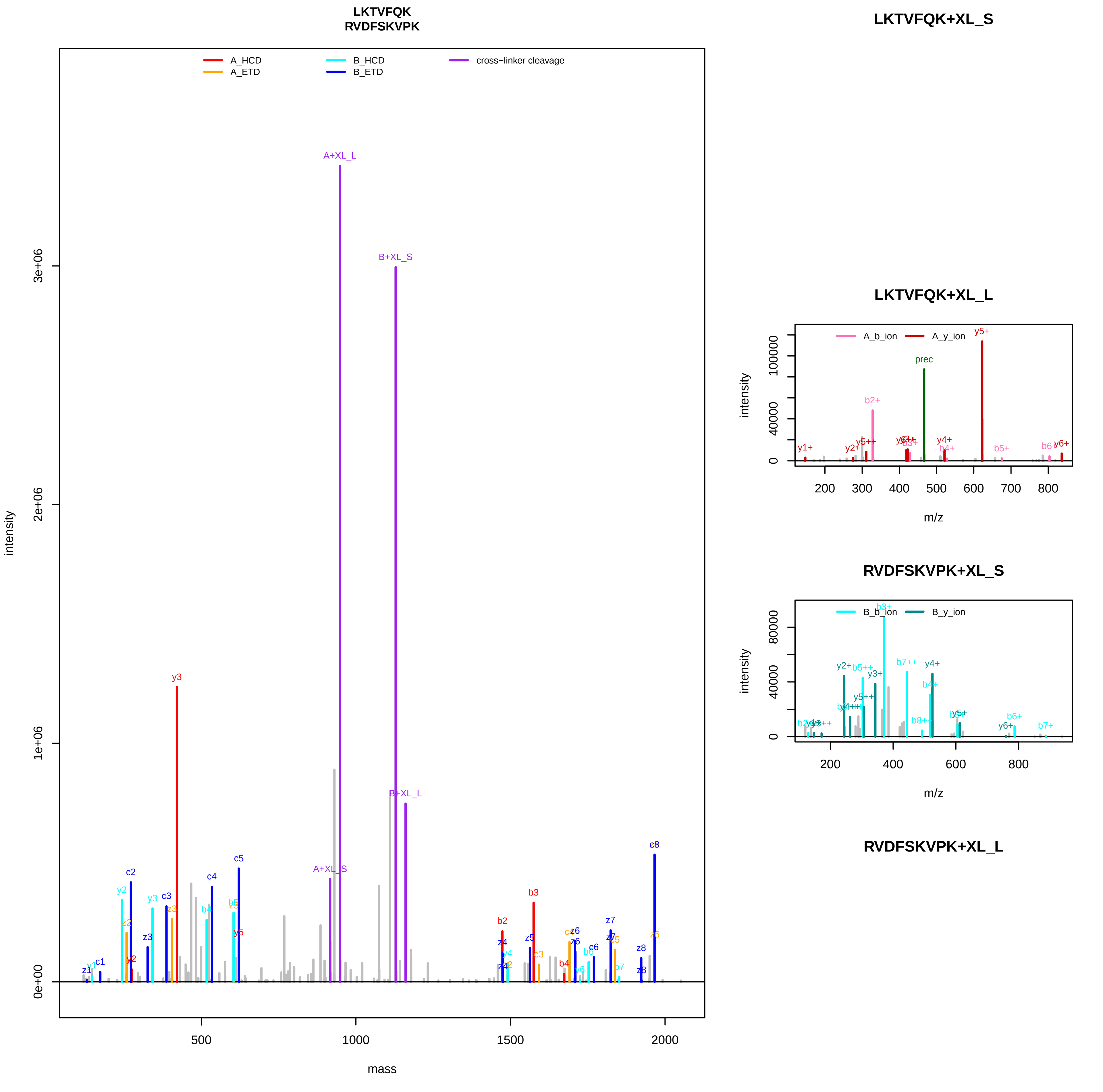
LKTVFQK
RVDFSKVPK
A_HCD
A_ETD
B_HCD
B_ETD
cross−linker cleavage
A+XL_L
B+XL_S
B+XL_L
A+XL_S
y3
b3
b2
b4
c2
c3
c4
c5
c6
c8
y2
z1
c1
z3
z4
z5
z6
z7
z8
intensity
0e+00
1e+06
2e+06
3e+06
mass
500
1000
1500
2000
LKTVFQK+XL_S
LKTVFQK+XL_L
A_b_ion
A_y_ion
prec
y5+
b2+
y1+
y2+
y4+
b5+
y6+
intensity
0
40000
100000
m/z
200
300
400
500
600
700
800
RVDFSKVPK+XL_S
B_b_ion
B_y_ion
b3+
y2+
y3+
b7++
y4+
b4+
y6+
b6+
b7+
intensity
0
40000
80000
m/z
200
400
600
800
RVDFSKVPK+XL_L
